# New Perspectives in the Use of Biomaterials for Periodontal Regeneration

**DOI:** 10.3390/ma12132197

**Published:** 2019-07-08

**Authors:** Federico Ausenda, Giulio Rasperini, Raffaele Acunzo, Angelina Gorbunkova, Giorgio Pagni

**Affiliations:** Unit of Periodontology, Department of Biomedical, Surgical and Dental Sciences, University of Milan, Foundation IRCCS C’a Granda, 20142 Milan, Italy

**Keywords:** periodontal biomaterials, periodontal regeneration, membranes, bone graft, scaffolds

## Abstract

Periodontitis is a disease with a high prevalence among adults. If not treated, it can lead to loss of teeth. Periodontal therapy aims at maintaining patient’s teeth through infection control and correction of non-maintainable anatomies including—when possible—regeneration of lost periodontal tissues. The biological regenerative potential of the periodontium is high, and several biomaterials can be utilized to improve the outcome of periodontal therapy. Use of different natural and synthetic materials in the periodontal field has been studied for many years. The main materials used today in periodontology analyzed in this review are: Resorbable and non-resorbable barrier membranes; autogenous, allogeneic, xenogeneic, and alloplastic bone substitutes; biological agents, such as amelogenins; platelet-derived growth factor; bone morphogenic proteins; rh fibroblast growth factor 2; teriparatide hormone; platelet concentrates; and 3D scaffolds. With the development of new surgical techniques some concepts on periodontal regeneration that were strictly applied in the past seem to be not so critical today. This can have an impact on the materials that are needed when attempting to regenerate lost periodontal structures. This review aims at presenting a rationale behind the use of biomaterials in modern periodontal regeneration

## 1. Introduction

Periodontal diseases are an array of inflammatory conditions usually triggered by a bacterial infection that affect the tissues surrounding the natural dentition (gingiva, connective tissue, periodontal ligament, cementum, and alveolar bone) [1]. Dental biofilm-induced gingivitis, non-dental biofilm-induced gingival diseases, necrotizing periodontal diseases, periodontitis, periodontitis as a manifestation of a systemic disease, systemic diseases or conditions affecting the periodontal supporting tissues, periodontal abscesses and endodontic-periodontic lesions, mucogingival deformities and conditions are some of the possible clinical manifestations of periodontal disease [2]. If left untreated, loss of periodontal support can continue at different rates over time [3].

The prevalence of these conditions is high—almost 50% of US adults aged ≥30 years are affected by periodontitis [4]. In other parts of the world such as Southern Europe, the prevalence of periodontal diseases is even higher. A recent investigation by Aimetti et al. showed that in the Turin area in Northern Italy, up to 75% of the population was affected by moderate to severe forms of periodontitis [5]. Severe forms of periodontitis are considered to be the sixth most prevalent disease of mankind [6]. The different forms of periodontal diseases may have different etiology or contributing factors, but they all lead to clinical attachment loss. Once the initial phase of periodontal therapy is completed, the etiological and contributing factors are controlled, and periodontal inflammation is no longer present, however, the loss of periodontal structures still remains. At this point the clinician is challenged with the decision to treat the deformities caused by the disease progression over time. The deformities can include horizontal bone defects, periodontal intrabony defects, furcation defects, and/or gingival recession (REC). The second phase of periodontal therapy (surgical phase) aims at correcting the deformities caused by the disease in order to facilitate oral hygiene maneuvers, reducing the possibility of disease recurrence and restoring the patient to optimal oral health [7].

An intrabony defect is defined as a periodontal defect surrounded by one, two, or three bony walls or a combination of these [8]. A furcation defect may manifest itself as pathological resorption within a furcation. Continual intrabony and furcation defects after periodontal phase I therapy increase the risk of tooth loss and negatively impact patients’ function and aesthetics. Various treatment modalities have been investigated to repair/regenerate periodontal tissues. The goal of periodontal regeneration is to gain periodontal attachment in severely compromised teeth, decrease pocket depth, and stabilize gingival recession [9].

The aim of this review is to present the scientific and clinical basis for the use of biomaterials in periodontal regeneration. 

## 2. Periodontal Regeneration and Biomaterials

When periodontal disease leads to attachment loss, the periodontal ligament (PDL) and the bundle bone are resorbed, and bacterial plaque can be found on the root surface [10,11]. If plaque and calculus are removed, a periodontal pocket may heal with the formation of a long junctional epithelium [12]. Several treatment options that can be utilized to treat periodontal disease include scaling and root planning, open flap debridement (OFD), guided tissue regeneration with barrier membranes (GTR), bone-replacement graft materials, and the use of biologically active regenerative materials [9]. Clinical studies showed the conjunct use of barrier membranes and biomaterials to be more effective in obtaining clinical attachment (CAL) gain and probing pocket-depth (PPD) reduction versus OFD alone. In two systematic reviews and meta-analysis by Murphy and Gunsolley [13] and Needleman [14], intrabony defects treated with GTR showed a CAL gain, PPD decrease, and REC increase more favorable than OFD alone. In another systematic review, Trombelli found favorable CAL and PPD changes and increased defect fill with GTR when compared with OFD alone [15]. These reviews highlight the great advantages of the use of biomaterials but also highlight the variability among studies and the difficulty in assessing the clinical relevance of the findings. 

Periodontal regeneration aims at the new formation of tooth-supporting tissues including cementum, PDL, and alveolar bone on a previously diseased root surface. The restitutio ad integrum of the lost periodontal structures is achieved through differential tissue responses. The work of Murray, Hurley, Boyne, Melcher, and Nyman and Karring helped discover the biologic principles of periodontal healing [16,17,18]. Melcher proposed the idea that the repopulation of cells on the root surface after periodontal surgery determined the nature of the attachment that will form [17]. After surgery and removal of biofilm, the root surface can be repopulated by epithelial cells, gingival connective tissue cells, bone cells, and periodontal ligament cells. Melcher’s idea was that the periodontal defect area would be first colonized by the fastest cells, which were epithelial cells. Nyman and Karring documented the ability to regenerate lost periodontal tissues in a human subject [18]. They positioned a Millipore filter between the flap and the previously diseased root, performing the first case of periodontal guided tissue regeneration. It was shown for the first time in humans that new cementum, PDL, and bone could be regenerated around a previously diseased root surface. Their conclusion was that through selective cellular infiltration, the cells that populate a wound area first determined the type of tissue that will ultimately occupy the space. With this knowledge, techniques and materials were utilized as barrier membranes to prevent undesired cells from accessing the wound while concurrently allowing the desired cells to access the wound space, leading to what is now known as guided tissue regeneration (GTR). Years later, the concept of GTR was applied also to regenerate bone for implants. Buser coined the acronym GBR [16]. The concept of GTR spread widely, and it is still used by several authors to explain the biological principles of periodontal tissue regeneration.

Further research switched the focus from the competition theory to the one of blood clot stability. The studies from Wikesjo [19,20] suggested that epithelial cells would not be able to migrate between the clot and the root surface as long as adhesion between the blot clot and the root was present, so that periodontal regeneration can be achieved. In other words, the competition theory is true, but fastest cells are not always able to migrate into a defect even when a barrier membrane is not used. A typical example of this is the healing of the extraction socket. This model has been thoroughly studied and evaluated. Even in the clinical setting, it is easily understood that the epithelium is not able to migrate within an alveolar socket and that the alveolus normally fills with bone instead of soft tissues. The work of Cardaropoli [21] showed that the epithelium only starts migrating over the alveolus once the granulation tissue starts replacing the blood clot, and it never goes beyond the level reached by the granulation tissue. The same is probably true even for the periodontal defects—when a blood clot is stable and the tooth mobility is low, the epithelium may have to wait for granulation tissue to form before starting to migrate into the defect.

With this concept in mind, surgical techniques have evolved from large flaps to smaller ones in the attempt to preserve the soft tissues as much as possible. Better clinical results were seen with minimally invasive surgical techniques that aimed at preserving the soft tissue and limiting the mobility of the flap as much as possible. The papilla preservation flap was first introduced by Takei in 1985 [22] and later modified by Cortellini [23,24,25]. In the following decade, other surgical approaches using a single flap were developed [26,27,28], and more recently, surgical solutions that combined the concepts learned from periodontal plastic surgery and periodontal regeneration in the attempt to maximize the regenerative potential of the periodontium have been described [29]. The evolution of regenerative techniques moving from large flaps to more and more minimally invasive ones opened the possibilities for multiple biomaterials to be utilized in order to regenerate periodontal tissues [30] while limiting the possibility of using others.

The main types of biomaterials that have been used in periodontal regeneration are: Barrier membranes, grafting materials, biological agents, and, more recently, 3D scaffolds.

### 2.1. Barrier Membranes

Barrier membranes were used with two main goals. The first one was to create a barrier between the soft and the hard tissues following the competition theory. We already discussed how this may or may not need to be revised in light of more recent concepts. The second one is the mechanical ability of a membrane to separate the forces applied to the soft tissues from the underlying graft augmenting the stability of the latter. Membranes exclude unwanted epithelial cells, provide space for appropriate cells (i.e., PDL cells, bone cells, and/or cementoblasts), and increase blood-clot stability in order to improve the outcome of periodontal regenerative procedures [9,31]. Their function is also that of supporting and stabilizing the underlying tissues to improve the clinical outcome of regenerative procedures.

Following the first attempts with the Millipore filter, non-resorbable expanded polytetrafluoroethylene (e-PTFE) membranes were developed either with or without a titanium reinforcement. These membrane allowed for optimal graft stabilization for as long as needed. Unfortunately, though, they required a second surgical intervention for the removal of the membrane, and flap opening often occurred, reducing the potential for periodontal regeneration. In time, resorbable membranes were developed, eliminating the need for a re-entry procedure, reducing the chance for flap opening and limiting the damage created by such a complication. More recently, an evolution of PTFE technology also reducing the damage created by membrane exposure revamped the use of non-resorbable membranes in bone and tissue regeneration.

Below you will find a list of the most commonly used non resorbable and resorbable membranes and their biological characteristics.

Classification:

1. Nonresorbable
  a. Cellulose acetate
  b. Expanded polytetrafluoroethylene (e-PTFE) with or without titanium reinforcement
  c. Dense polytetrafluoroethylene (d-PTFE)
  d. Titanium-reinforced high-density polytetrafluoroethylene (Ti-d-PTFE)
2. Resorbable
  a. Natural
  b. Synthetic

Nonresorbable: Historically, the first membrane utilized for GTR was a bacterial filter made from cellulose acetate (Millipore®) [17]. However, due to its poor clinical application, the e-PTFE membrane was developed, and the first generation of documented clinical studies utilized it for periodontal regeneration. A synthetic polymer, e-PTFE has a porous structure that allows tissue ingrowth. PTFE is exposed to high tensile stresses to expand and to create a porous microstructure. Characteristics of e-PTFE are its biocompatibility and resistance to enzymatic degradation by the host and microbes [32]. A nonporous synthetic polymer is d-PTFE that does not allow ingrowth of tissue.

Barrier membranes used alone and not in conjunction with particulate graft materials may be subjected to membrane compression and/or collapse into the defect space due to overlying pressure from the soft tissue. When clinical cases require larger areas of space maintenance, a more rigid structure is required. To overcome these challenges, a titanium-reinforced high-density polytetrafluoroethylene (Ti-d-PTFE) or a titanium framework placed in between two layers of e-PTFE may be utilized [33]. The integration of titanium provides a non-resorbable, biocompatible material with high strength and rigidity, resistant to corrosion for the purpose of increasing mechanical stability, maintaining a larger area of space and preventing the collapse of the barrier membrane. Nonresorbable membranes of e-PTFE have been used successfully in animal experiments and in several clinical studies [9,34,35,36,37] and have shown evidence of periodontal regeneration. These materials are unable to integrate with the surrounding tissue. Although providing structural integrity for regenerative therapy, a second re-entry procedure is required to retrieve the membrane. The second surgery increases the risk of site morbidity, and regenerated tissues are more susceptible to iatrogenic damage and bacterial contamination. If during healing, a membrane is exposed prematurely, there is an increased rate of soft tissue complications: Dehiscence and infection. Once exposed to the oral cavity and microbes, the porous surface of e-PTFE membranes is rapidly colonized leading to a higher risk of infection and untimely removal of the membrane and disruption of regeneration [38]. For these reasons, clinical research and clinicians moved towards resorbable membranes.

Resorbable: To overcome the shortcomings of nonresorbable barrier membranes, advancements of a barrier membrane with biodegradable properties evolved. Resorbable barrier membranes are fabricated from either native collagen (natural) or aliphatic polyesters (synthetic), such as polylactic acid or polyglycolic acid [30].

Natural resorbable barrier membranes are fabricated with collagen from either human or animal tissue. Collagen membranes allow for good tissue integration, fast vascularization, hemostasis, and chemotaxis for periodontal ligament fibroblasts and gingival fibroblasts [29]. A major advantage over nonresorbable barrier membranes is that resorbable membranes do not require an additional surgery for membrane removal, therefore decreasing patient morbidity, time, and cost. Studies completed by Johns and Wang [39,40], later validated also by the findings of Schlegel [41], confirmed collagen membranes lack immunogenicity and therefore will not create a foreign body reaction. Unlike nonresorbable membranes, collagen membranes stimulate spontaneous healing in the event of a mucosal dehiscence [36]. The exposed collagen will slowly epithelialize through secondary wound closure, reducing the risk of infection, additional surgical intervention, and interruption to the regenerated site [42].

Limitations of collagen membranes include poor mechanical properties and therefore susceptibility to collapse and loss of space-maintaining ability. A major obstacle that resorbable membranes face is the unpredictable resorption time and degree of degradation. The resorption of collagen membranes is dependent upon the source of material (bovine, porcine, human) and the breakdown rate of collagen into oligopeptides and amino acid molecules. Collagen membranes are absorbed through enzymatic degradation by collagenases/proteases and macrophage/polymorphonuclear leukocyte-derived enzymes and bacterial proteases [42]. Depending on the material of the resorbable membrane, the timing of degradation may be faster than the wound healing process, decreasing the stability of the barrier causing early exposure to unwanted epithelial cells.

Various biological, chemical, and enzymatic processes have been developed to prolong the breakdown of the collagen membrane through cross-linking. Most commonly used chemically are glutaraldehyde, 1-ethyl-3-(3-dimethylaminoprophyl) carbodiimide, polyepoxy, diphenyl-phosphorylationazide [43]. Within multiple animal and clinical studies, authors concluded that the higher the degree of cross-linking the slower the rate of degradation of the collagen membrane [31]. However, cross-linked collagen membranes come with precautions. One cross-linking technique, glutaraldehyde technique, has been reported to leave cytotoxic residue during the process [31].

A question still needed to be answered is what the ideal degradation time would be for a resorbable membrane. Bottino et al. [44] suggest that regardless of the resorbable or nonresorbable nature of the membranes, these devices must not degrade and must function for at least 4–6 weeks to allow successful regeneration of the periodontium. As a resorbable membrane does not need to be removed, it may be stated that the longer it maintains its space maintenance ability the better. On the other side, Wikesjo’s work showed that the game is mostly played in the first few weeks. Already in the first 24 h, a stable clot is created that will be replaced by granulation tissue in the first few days. A bone matrix completely replaces the granulation tissue in 2–4 weeks. The bone matrix will mature into woven bone first and then lamellar bone. With this knowledge in mind, we can safely state that after a few weeks the role of the membrane may be limited, moreover the persistence of a membrane may reduce the vascularity from the flap side, reducing the speed of the maturation phase, as seen in some experimental studies [45].

### 2.2. Grafting Biomaterials

Numerous bone grafting materials are available for the clinician today and have been used to achieve periodontal regeneration or alveolar ridge reconstructions. In the United States alone, almost 150 different types of bone grafts are available on the market [46]. Hard tissue replacement materials for periodontal regeneration are categorized into one of four categories: Autogenous bone; allogeneic bone substitutes, such as freeze-dried bone allograft (FDBA) and demineralized freeze-dried bone allograft (DFDBA); xenogeneic; and alloplastic. Clinically, these materials are used for supporting the soft tissues from collapsing into the defect and for their ability to stabilize the clot and facilitate bone formation.

Autogenous bone is harvested from a donor site in the same individual and transplanted to another site. Autogenous bone has been considered the gold standard because it acts as scaffold, and it has osteoconductive, osteoinductive, and osteogenic properties [46,47]. It has no potential complications of histocompatibility [48]. Its use has been studied to fill intrabony defects and to regenerate bone in edentulous areas. In the oral cavity it is usually harvested from surrounding alveolar bone with scrapers from the tuberosity, from the ramus, or from the retromolar region or from the symphysis region in the mandible. As the bone needed for periodontal regeneration is limited, extra oral sites are not usually considered. Its use is not very popular nowadays because its biological advantages are to be weighted with the biological cost of harvesting bone from a second donor site, leading to higher morbidity, increased surgical time, and risk of graft contamination. Moreover, its replacement rate may be unpredictable [49,50].

Allogeneic bone substitutes are materials that are derived from a human donor (same species) with a different genetic heritage. Different forms exist: Fresh-frozen (FFBA), FDBA, and DFDBA. They lead to less patient morbidity, since they overcome the disadvantage of the need for a second surgical site from which to harvest the graft, and if needed a large quantity can be used. Allogeneic bone substitutes have demonstrated good osteoinductive capabilities. DFDBA has also shown limited osteoinductive capabilities. It can be found as cortical wedges, cortical chips, cortical granules, cancellous powders prepared as froze, freeze dried, mineralized and demineralized bone [51]. FFBA provides the highest osteoconductive and osteoinductive potential among all allograft materials, though it is no longer used due to the risk of disease transmission [51,52]. Allogeneic bone freezing and drying processes are performed in order to reduce antigenicity. FDBA is osteoconductive and, mixed with autografts, is thought to be the best combination to enhance osteogenic potential. The freeze-drying process distorts the 3D presentation of the human leukocyte antigens on the surface of graft particles that affects the immune recognition. FDBA is more slowly resorbed than DFDBA [53]. Demineralized freeze-dried bone allografts have a potential for osteoinduction with some expression of bone morphogenetic proteins (BMP). Some authors state that DFDBA has osteoinductive properties [54,55], others say that its osteoinductive potential is low [55,56,57]. Its complete replacement by new autologous bone is slow [58].

Xenogeneic bone substitutes are grafting materials that derive from a different species than that of the receiving organism. Xenogeneic scaffolds available for periodontal regeneration can be of bovine, porcine, or equine origin. Like allografts, they spare the patient the need for a second surgical site, leading to less morbidity. These biomaterials undergo a deproteinization and demineralization process through thermal and chemical treatment with the utilization of sodium hydroxide. These processes may not be efficacious against the elimination of prions that may be responsible for diseases such as bovine spongiform encephalopathy and Creutzfeldt–Jakob disease [59,60]. For these reasons, some of the main industries that produce these materials utilize only animals from countries where these diseases have never been found. The prions that act as carriers for this diseases are found in the medullary tissues, for this reason only the tissues located far away from the medullary tissues are processed. After the deproteinization process, what is left is the mineral component, which is made of calcium phosphate and calcium carbonate in the form of a reticulum made of apatite crystals. These networks help blow clot stabilization and bone apposition. Different companies use different manufacturing processes that have an impact on the wettability and the surface characteristics of the material. Xenografts can usually be found as cortical or cancellous particles with different granule sizes or as cancellous blocks. They have good osteoconductive properties [61,62] and a slow resorption rate. Long-term histological analyses found evidence of xenogeneic particles from as late as three years after implantation [63] to as late as 16 years [64].

Alloplastic bone substitutes are grafting materials for periodontal regeneration. They are synthetic products that provide no risk of infections and are easily available, biocompatible, and have osteoconductive properties [65]. They can be made of several different materials. Bioceramics have a similar structure to the inorganic bone component [66]. Absorbable/non-resorbable hydroxyapatite is biologically inert and biocompatible. It acts as a filler, does not contribute to bone formation, and has a slow resorption rate [67]. Beta-tricalcium phosphate (β-TCP) once placed is completely resorbed in six to nine months and substituted by new bone [68]. Bioglass materials are made of a glassy ceramic, zinc oxide, and calcium oxide, they also have osteoconductive properties but are hardly resorbed. Biocoral allografts are mostly made of calcium carbonate in the form of argonite or pure calcium carbonate, strontium, fluoride, magnesium, sodium, potassium. Biocoral is an osteoconductor and allows bone growth both by apposition and by substitution [69,70,71]. Polylactic acid polymers are also available for use in periodontal regeneration. They are biocompatible and biodegradable [71]. The bioproducts are potentially toxic but are released in such small amounts that they have no dangerous effects.

Given the large array of grafting biomaterials available, the clinician may find him/herself lost in the selection of the best option. With the exception of DFDBA, which may retain some osteoinductive properties, most grafting materials exert their function through their osteoconductive properties. In contemporary periodontology, usually autogenous bone is not the grafting material of choice for periodontal regeneration since its harvesting often requires a second surgical site and its resorption rate is unpredictable [50]. Allogeneic bone substitutes, FDBA, and DFDBA are considered the gold standard. FDBA is usually more slowly resorbed than DFDBA [54,55]. These allogeneic materials do not require a second surgical site, they promote regeneration by stabilizing the blot cloth and preventing the soft tissue collapse into the defect. The same properties are peculiar to xenogeneic grafting materials too. They act as osteoconductive materials, providing a scaffold for stabilization and organization of the blood cloth, they do not require a second surgical site, and they are characterized by a slow resorption rate. In fact, there is evidence showing their presence as late as 16 years after implantation [64]. Some patients for personal or religious reasons may refuse to undergo treatment that entails the utilization of xenogeneic or allogeneic materials. An option to overcome this limitation may be the use of alloplastic grafting materials. These promote osteoconduction [65] and are slowly resorbed [68].

One may argue that the utilization of xenogeneic or alloplastic materials does not lead to “true” periodontal regeneration, since years later there will still be histological evidence of non-resorbed graft particles. In these sense, only autogenous or allogeneic grafting materials should be used to achieve periodontal regeneration. However, from a clinical standpoint, successful periodontal regeneration can be achieved also with the use of xenogeneic or alloplastic options.

### 2.3. Biological Agents

Membranes and scaffolding graft materials have been used to take advantage of their osteoconductive properties. Several biologic agents have been studied to stimulate osteoinduction. The concepts behind the use of growth factors and differentiation factors in oral tissue regeneration are based on the seminal research by Marshall R. Urist in the late 1960s [72,73], but it was not until the late 1980s to early 1990s that the use of growth factors started to be tested directly for periodontal regeneration [74,75]. Thanks to modern technology, growth factors can be produced increasing the concentration of the original molecule by thousand- or million-folds. When the initials *rh* are found before the name of the molecule it means that a recombinant (r) technology has been used to amplify the human (h) version of that protein. Today several growth factors have been investigated for their use in the oral cavity to regenerate hard and soft tissues. For the purpose of this review the focus will only be placed on the growth factors that have been cleared by the United States Food and Drug Administration for periodontal regeneration.

Amelogenins: One of the most widely used biologics is enamel matrix derivative (EMD). It was first introduced to the market in the late 1990s, and it is an extract of porcine fetal amelogenin used to stimulate periodontal regeneration to form new bone, PDL, and cementum. EMD is thought to mimic the development of the tooth-supporting apparatus during tooth formation [76]. Clinical and histologic studies have demonstrated that EMD will regenerate the periodontal attachment apparatus on teeth previously affected by periodontal disease in advanced periodontal defects [77]. Some clinical investigations have not found great clinical improvements with the use of EMD [78], however, there is evidence of regeneration with the use of EMD both in furcation [79] and in intrabony defects [80]. In a systematic review, Esposito found that EMD significantly improved CAL by 1.1 mm and reduced PPD by 0.9 mm [80].

Platelet-derived growth factor: Recombinant human platelet-derived growth factor (rh-PDGF) can be used to treat intrabony defects and gingival recession deformities. It is manufactured using recombinant DNA technology, and it is mitogenic and chemotactic for osteoblasts, cementoblasts, and PDL cells. Its clinical use in conjunction with a carrier (β-TCP or DFDBA) has been investigated with clinically positive results [81,82,83]. In a human study testing the use of rh-PDGF along with DFBA in intrabony defects, Nevins [81] found that the test group had statistically significant CAL gain of 6.17 mm, PPD reduction of 6.42 mm and radiographic bone fill of 2.14 mm compared to the control. In a large multicenter trial where rh-PDGF was used along with β-TCP as carrier at concentrations of 0.3 mg/mL, 1.0 mg/mL, or a buffer control. Three months after surgical intervention it was found CAL gain and bone fill were greater for the group that received the lower concentration rh-PDGF versus the control group. Darby and Morris [84] conducted a systematic review and found that at six months after use of rh-PDGF and β-TCP as carrier, both CAL gain and radiographic defect fill were in favor of the test group.

Bone morphogenic proteins (BMPs): The most commonly studied BMPs in the periodontal field are BMP-2 and BMP-7. They have osteoinductive potential as they have the ability to stimulate PDL cells differentiation into osteoblasts and increase expression of mineralized tissue markers [47,85,86]. RhBMP-2, along with a collagen sponge as carrier, has been successfully used for ridge preservation procedures following tooth extraction in both molar areas [87,88] as well as in larger defects [89]. Its utilization in intrabony and furcation defects has been successful [90], however, ankylosis and root resorption has also been described [91]. RhBMP-2 has also shown promising results when used for sinus augmentation [92] with histological data suggesting similar bone quality and quantity between grafted and autogenous bone [93]. When rhBMP-2 is utilized in combination with autologous bone it appears to further enhance cell activity [94]. BMP-7 has been studied delivered with a collagen sponge to stimulate bone growth in sinus augmentation procedures. In a study comparing its use against deproteinized bone alone, there was a significant higher amount of newly formed bone in the control group [95]. It is interesting to notice that not all BMPs function in the same way—Wikesjo compared rhBMP-2 and rhBMP-12 in a canine model, rhBMP-2 resulted in more robust bone formation, but periodontal fibers run parallel to the root surface, while rhBMP-12 resulted in perpendicular finer orientation [96]. BMPs appear to have good potential and can be utilized as alternatives to bone grafts for ridge preservation or for sinus augmentation. More evidence is needed to understand indications, dosage, and carrier optimization.

Rh Fibroblasts Growth factor 2: RhFGF-2 is a protein of the FGFs family. It has been shown to promote bone formation in vivo [97,98]. In an animal model, local application of FGF-2 significantly increased periodontal regeneration in class II furcation defects compared to control sites [99,100]. In humans, FGF-2 use has been investigated by Kitamura et al. [101]. The results of their investigation showed that the percentage of bone fill in FGF-2-treated sites administered at different concentrations compared to vehicle alone was significantly greater at 36 weeks, with no adverse effects. Currently, the effect and proper dosage of FGF-2 still needs to be better understood.

Teriparatide hormone: It is a biologic agent made up by the first 34 amino acids of parathyroid hormone’s (PTH). It is commonly used for the treatment of osteoporosis. It has been shown to have an effect of PDL cell survival and on the expression of osteoprotegerin [102,103,104]. Surgical treatment of intrabony defects along with daily injections of Teriparatide PTH and vitamin D supplement showed clinical attachment levels gain [105]. In a feasibility study in which after implant placement the test group received daily injections of Teriparatide, it was found that the treated groups had implants that showed a higher bone to implant contact in the periosteal and medullary portions compared to the control group [106]. At the present moment, this biological agent has shown some positive short-term effects, however, larger and longer investigations are needed to better understand its clinical applications.

Platelet concentrates: Platelets are indispensable for hemostasis and are a source of growth factors. Platelet’s alpha granules contain platelet-derived growth factor (PDGF), vascular endothelial growth factor (VEGF), insulin-like growth factor (IGF), and transforming growth factor-beta (TGF-*β*) [107]. Different platelet concentrates, such as platelet-rich plasma (PRP), pure platelet-rich plasma (P-PRP), leukocyte- and platelet-rich plasma (L-PRP), platelet-rich fibrin (PRF) have been used in periodontology. The use of these biologics has been investigated as a substitute for connective tissue in sinus grafting procedures and as a barrier membrane for periodontal regeneration [108,109,110,111]. When used for recession coverage as a substitute for a connective tissue graft or as a substitute for barrier membranes in GTR, the clinical results were found not to be superior to conventional therapy [111,112]. The great variability of study designs, concentrations, techniques, and outcomes makes it difficult to compare data and reach conclusions. At this moment, platelet-derived concentrates have shown to have a positive effect on soft tissue healing therefore indirectly also on bone growth. More studies are needed to understand the biological mechanisms of platelet-derived concentrates and their clinical use.

Various growth factors and/or signaling molecules are available for periodontal regeneration. Some of these agents, such as EMD, have been used for decades now. Others are still relatively new, and their mechanism of action is yet to be fully understood. EMD is certainly the most routinely used biological agent in periodontal regeneration. More basic science and clinical research is needed to have a better understanding of the biological mechanisms, the long-term effects, and the utilization of the proper carrier and ideal concentration of these agents.

### 2.4. 3D Scaffolds

The possibility of regenerating lost periodontal structures is limited by the defect anatomy, and its potential is proportional to the defect angle [112,113] and to the number of bony walls [114]. The biggest clinical challenge for the surgeon is often that of obtaining intimate adaptation of a block graft with the receiving bed. In the attempt to overcome this difficulty, new printable biomaterials are being studied so that they can be used to precisely fit in the defect evaluated through a conical beam computerized tomography. This type of technologies has been available for several years now but is not yet popular in daily practice. This may have to do to the fact that not only does the macrostructure of the 3D scaffold has to precisely adapt, but also its internal microstructure needs to have good adaption so that neoagiogenesis, cell migration from the neighboring tissues, and diffusion of nutrients into the tissue can lead to regeneration inside the graft. These characteristics make the definition of biomimetics, so that adhesion, cellular proliferation and cellular differentiation can lead to the formation of new tissues [115]. The optimal biomimesis is obtained through surface absorption of proteins that has to do with the characteristics of the materials that the scaffold is made of, in particular, how hydrophobic/hydrophilic the materials are, their surface roughness, superficial structure and porosity [116,117,118,119]. 

The materials that seem to show the best potential in oral and periodontal regeneration are hydrogels, nano fibrous scaffolds, nano/micro spheres, and multiphase scaffolds. In the periodontal field, the latter ones seem to be the most adequate because of the need to regenerate different tissue (bone, PDL, cementum). Multiple fabrication methods for 3D scaffolds exist. Traditional techniques are: Particle leaching, gas foaming, freeze drying, phase separation, fiber meshes/fiber bonding, melt molding, and solution casting [120]. Their main disadvantage is that these techniques do not allow for control of dimension, geometry, interconnectivity, and uniformity of the disposition of the internal pores. Methods such as electro-spinning or self-assembly do solve this problems but are complex and do not grant an adequate final mechanical stability. The solid free-form technique, also known as rapid prototyping, appears to be the most appropriate one. It can be done through fused deposition modeling, selective laser sintering, or stereolithography, and it is compatible with materials such as artificial polymers, ceramics, and metals [121].

Recent use of these biomaterials has been investigated to regenerate new bone and new PDL. In the periodontal field, the 3D scaffold is custom made after analysis of the defect that has to be treated. The component of the scaffold designed for bone regeneration is made of polycaprolactone and has a trabecular internal architecture with communicating pores that allows for the insertion of growth factors. The periodontal component of the scaffold is made of polylactic-polyglycolic acid and has cylindrical micro channels with an architecture that allows deposition of fibers. It has a thickness of 250 microns as that of the human periodontal ligament. Polylactic-polyglycolic acid is quickly resorbed, while polycaprolactone takes over one year’s time to resorb. The precision of the adaptation of the graft to the defect has on average a 96% adaptation ratio. The use of this material on different animal models has shown successful bone and periodontal regeneration [122,123].

As of today, there is one case report on the use of this technology in humans [124]. A 3D scaffold was used to treat an anatomically unfavorable periodontal defect on the mesiobuccal aspect of a lower left canine of a patient with periodontitis. The 3D scaffold was stabilized using resorbable polylactic acid pins and was infused with rh-PDGF and the patient’s own blood. The graft healed submerged for 12 months, and at the 13th month, part of it got exposed, perforating the soft tissue and leading to infection of the scaffold. The removal of the scaffold was necessary, this allowed for histological analysis which showed presence of small portions of new bone in contact with the residual polycaprolactone portions of the scaffold [124]. The lesson learned from this experiment is that polycaprolactone’s resorption rates are longer in the oral cavity than in other areas of the body. In the future, materials with faster resorption rates should be evaluated, or polycaprolactone should be stabilized with long-lasting devices, such as titanium screws.

## 3. Surgical Techniques and Biomaterials

**Figure 1:**
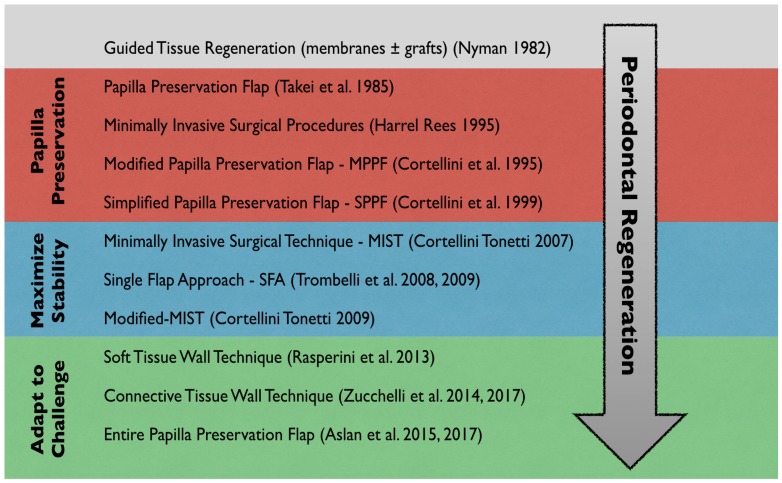
Evolution of flap designs for periodontal regeneration in relation to biological and clinical concepts.

Since the birth of periodontal regeneration [17,18], the use of biomaterials has evolved along with the evolution of the surgical techniques and flap designs. Initially, the combination of extended flaps along with the utilization of barrier membranes and bone grafts was the treatment of choice [18]. Then preservation of soft tissue, with particular attention to the interdental papilla, became the focus of clinicians. The papilla preservation flap first introduced by Takei [22] and then modified by Cortellini [23,24] was developed with the aim of preserving tissue in the interdental area to favor wound healing and to minimize the risk of secondary intention healing. More recently, novel techniques like the entire papilla preservation flap (EPP) have been developed to completely preserve the interdental papilla in deep and wide intrabony defects [125,126]. Clinically it became clear that incisions had to be designed with the goal of reducing flap mobility in order to facilitate blood cloth stabilization minimizing invasiveness. These concepts were first applied in the mid-1990s by Harrel and Rees [127] and later further developed by other researchers like Cortellini with the minimally invasive surgical technique (MIST) [128] and its modification, the modified minimally invasive surgical technique (modified-MIST) [28] and Trombelli with the single flap approach (SFA) [26,27]. Traditionally, periodontal regeneration has always been limited by the anatomy of the defect. Today surgical techniques (such as the soft tissue wall technique [29]) that borrow concepts from periodontal plastic surgery have been proposed to try to overcome these limitations in the attempt to achieve supracrestal regeneration. Figure 1 summarizes the evolution of flap designs for periodontal regeneration.

**Figure 2:**
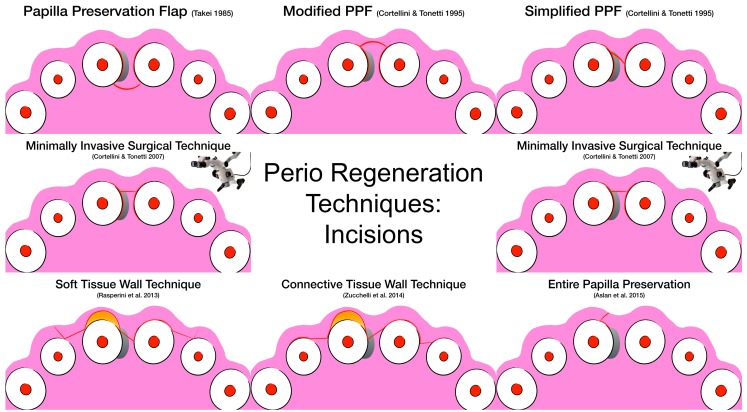
Visual representation of the surgical incisions according to surgical technique.

**Figure 3:**
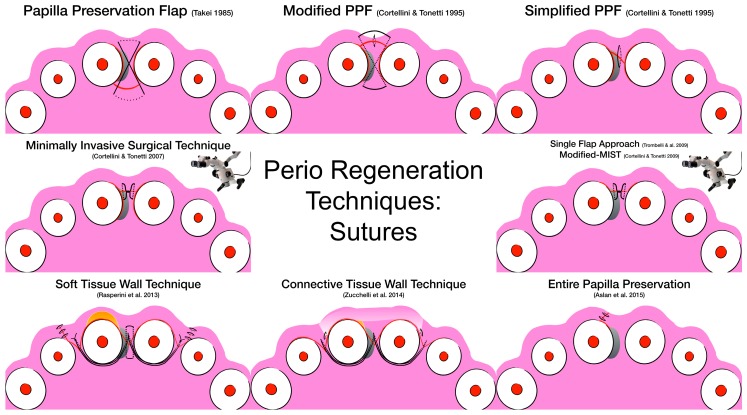
Visual representation of the suturing technique according to surgical technique.

This multitude of different surgical techniques, incision designs, and suturing techniques may create confusion among practitioners. Figure 2 and Figure 3 aim at visually describing the incision design and the suturing technique, respectively.

**Figure 4:**
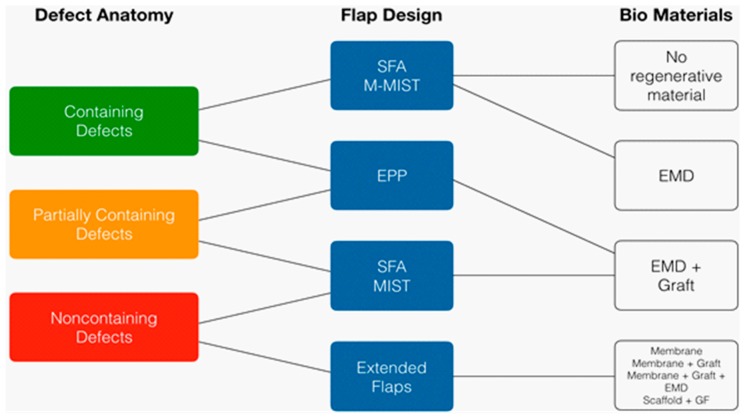
Choice of flap design and biomaterials according to defect morphology. SFA: Single flap approach, M-MIST: Modified minimally invasive surgical technique, EPP: Entire papilla preservation, MIST: Minimally invasive surgical technique.

Consequently, the traditional dogma according to which periodontal regeneration could only be achieved through GTR and the utilization of a barrier membrane and bone grafts seems not to be so true anymore. Today the greater attention is put on the non-surgical phase, and the surgical technique and biomaterials are used to support the soft tissues to avoid collapse into the defect and to stabilize the clot. A diagram of surgical technique and biomaterial according to defect type is presented in Figure 4.

## 4. Discussion

Today many biomaterials are available to use for periodontal regeneration. The interest of the researchers and the support from the companies allow for an enormous amount of clinical and pre-clinical research to be carried out every day. However, it is not possible to state what the best membrane, best bone graft, or best biologic agent is overall. There is no consensus on what biomaterial or combination of biomaterials offers the best performance for periodontal regeneration simply because each defect represents a different challenge, and every different anatomy may benefit from a different biomaterial or combination.

Regeneration is defined as the formation of new alveolar bone, new cementum, and new functionally oriented periodontal ligament. However, repair is a healing mechanism that results in a long junctional epithelium or in periodontal ligament fibers that run parallel, not perpendicular, to the root surface. Traditionally, since the first era of periodontal regeneration and the development of the concept of guided tissue regeneration, many clinicians and researchers think that true periodontal regeneration can only be achieved with the use of a bone graft and a barrier membrane to allow for epithelial exclusion. This is not always the case, in fact, while the clinical outcome of a GTR procedure may be very successful from a clinical standpoint with CAL gain, PPD reduction, and defect fill, the histological evidence may be lacking. As shown by Stavropolous and coworkers [129] in a clinical study in humans, periodontal defects treated with GTR with the use of a bovine bone graft and a bioresorbable membrane healed successfully from a clinical standpoint, but histological analysis of the teeth showed little evidence of new bone and new cementum but with collagen fiber running parallel to the tooth surface instead of perpendicular, therefore showing no complete true periodontal regeneration but only partial regeneration. The probable outcome of many clinically successful regenerative procedures is regeneration of the most apical portion of the defect and repair in the coronal part.

Given these findings, the concept of epithelial exclusion may have to be revisited, as suggested also by the model of a healing extraction site as stated above [21].

Irrespective of the quality of the histological findings, what is relevant for the patient and for the practitioner is clinical periodontal regeneration, which can be achieved as the successful result of a fruitful alliance between a compliant patient and a skilled periodontist. High levels of oral hygiene and complete control of inflammation are necessary, but the periodontal regenerative potential is still defined by the defect anatomy. Often this can be challenging, in fact, both on human skulls [130] or intrasurgically [131], investigators have found the majority of periodontal defects to have unfavorable anatomies.

With the evolution of periodontal surgery moving from large flaps to minimally invasive ones, the importance of biomaterials is still critical in large non-contenitive defects but seems to be less important in small intrabony ones. In particular, the need for a membrane may not be so critical anymore when treating contenitive defects with minimally invasive techniques, which, in any case, being so limited in term of flap reflection, would not even allow for membrane insertion. 

Minimally invasive techniques with very limited flaps have shown great results. The development of custom-made 3D scaffolds along with the use of growth factors is not yet sufficiently predictable for daily clinical application but it is very promising and may expand the horizons of periodontal regeneration beyond those defined by the defect anatomy. 

However, clinicians should be careful when judging these findings, since many clinical studies have showed improved clinical outcomes, but the healing processes behind these clinical studies remain not well understood.

## 5. Conclusions

Periodontal regeneration has evolved through different concepts, philosophies, and biomaterials. The improvement of clinical results are encouraging, but the lack of a complete understanding of the mechanisms lying behind tissue regeneration call for the development of new basic science models. Nevertheless, it is always important to keep in mind what the real goals of periodontal therapy are, namely the preservation of patients’ dentition in function, health, and aesthetics in the most efficient way so not to negatively interfere with patients’ quality of life.
